# Amplification of a *Zygosaccharomyces bailii* DNA Segment in Wine Yeast Genomes by Extrachromosomal Circular DNA Formation

**DOI:** 10.1371/journal.pone.0017872

**Published:** 2011-03-10

**Authors:** Virginie Galeote, Frédéric Bigey, Emmanuelle Beyne, Maite Novo, Jean-Luc Legras, Serge Casaregola, Sylvie Dequin

**Affiliations:** 1 INRA, UMR1083 Sciences Pour l'Œnologie, Montpellier, France; 2 CIRM-Levures, INRA, UMR1319 Micalis, AgroParisTech, Thiverval-Grignon, France; California State University Fullerton, United States of America

## Abstract

We recently described the presence of large chromosomal segments resulting from independent horizontal gene transfer (HGT) events in the genome of *Saccharomyces cerevisiae* strains, mostly of wine origin. We report here evidence for the amplification of one of these segments, a 17 kb DNA segment from *Zygosaccharomyces bailii,* in the genome of *S. cerevisiae* strains. The copy number, organization and location of this region differ considerably between strains, indicating that the insertions are independent and that they are post-HGT events. We identified eight different forms in 28 *S. cerevisiae* strains, mostly of wine origin, with up to four different copies in a single strain. The organization of these forms and the identification of an autonomously replicating sequence functional in *S. cerevisiae*, strongly suggest that an extrachromosomal circular DNA (eccDNA) molecule serves as an intermediate in the amplification of the *Z. bailii* region in yeast genomes. We found little or no sequence similarity at the breakpoint regions, suggesting that the insertions may be mediated by nonhomologous recombination. The diversity between these regions in *S. cerevisiae* represents roughly one third the divergence among the genomes of wine strains, which confirms the recent origin of this event, posterior to the start of wine strain expansion. This is the first report of a circle-based mechanism for the expansion of a DNA segment, mediated by nonhomologous recombination, in natural yeast populations.

## Introduction

The transfer of genetic information across normal mating barriers, also known as horizontal or lateral gene transfer (HGT), has long been recognized as one of the major forces driving prokaryote evolution, but has generally been seen as more limited in eukaryotic genomes [1], [2]. The increasing number of available genomic sequences has recently transformed this vision, as numerous cases of HGT have emerged in a wide variety of eukaryotic lineages (reviewed in [1], [3]). Several studies have reported the HGT of sequences of bacterial origin to the genome of *S. cerevisiae* [4], [5], [6], [7]. Introgressions between closely related yeast species [8], [9] or between varieties of the basidiomycete yeast *Cryptococcus neoformans* [10], [11] have also been described, suggesting that this mechanism is more widespread than previously thought in yeast genomes.

We recently reported the occurrence of HGT between more distantly related yeast species. The genome sequence of the commercial wine yeast strain EC1118 contains three gene clusters resulting from horizontal transfers [12], two of which are widespread among wine yeasts. The genes in these regions encode proteins involved in key metabolic functions during winemaking [12], [13], strongly suggesting that HGT is one of the mechanisms by which wine yeast strains adapt to their high-sugar, low-nitrogen environment.

The donor of one of these introgressions (region B, 17 kb) was identified as *Zygosaccharomyces bailii*, a major contaminant of wine fermentations, supporting the view that genetic exchange is favored by ecological proximity. The sequences of region B and of the homologous region in *Z. bailii* are almost identical (99.7%), suggesting that this introgression event is recent. However, despite the high degree of sequence similarity, differences in gene organization were found between the *Z. bailii* and EC1118 sequences, suggesting that some reorganization occurred after the transfer [12]. The mechanisms underlying the acquisition and reorganization of this DNA fragment have yet to be elucidated.

We report here the presence of multiple copies of the *Z. bailii* DNA segment in the diploid genome of EC1118, inserted into different chromosomes and displaying changes in structural organization. A broader survey of this region in the European yeast population revealed the presence of up to eight different forms, mostly in wine yeast strains. The structural organization of this sequence, the presence of a functional ARS and an analysis of the insertion breakpoints strongly suggested an expansion mechanism involving the formation of an extrachromosomal circle DNA (eccDNA) molecule and its integration into the yeast genome by nonhomologous recombination.

## Results

### Identification of three copies of region B in the genome of EC1118

**Figure 1:**
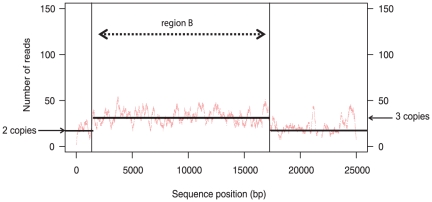
Estimation of the number of copies of region B in the diploid genome of strain EC1118. The number of sequencing reads covering a nucleotide position was used to identify changes in sequence copy number. The diploid chromosomal region, present in two copies (left side arrow), has 2n = 18 reads, whereas region B was found in three copies (arrow on the right) with 3n = 27 reads.

**Figure 2:**
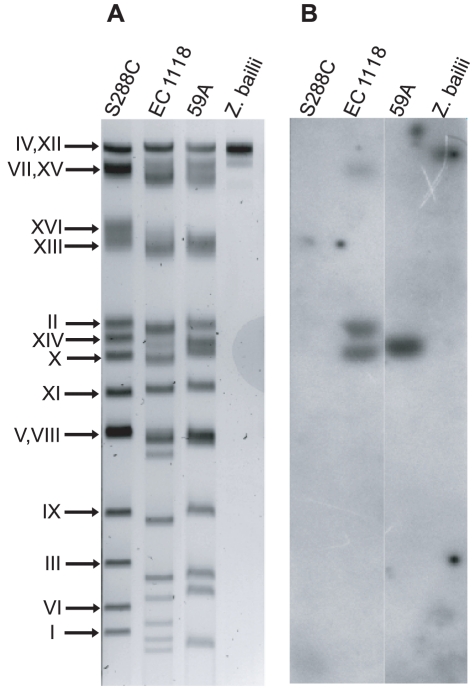
Chromosomal location of region B in different strains by Southern blot hybridization of PFGE gels. A. Chromosome separation by PFGE. Chromosomes were numbered according to the *Saccharomyces cerevisiae* nomenclature. B. Hybridization of a PFGE blot with a region. B. specific probe (gene 0023g). Positive signals were detected on chromosomes XII, XIV and X for the diploid strain EC1118 and only on chromosome X for the meiotic spore 59A

In the genome sequence of strain EC1118, we previously identified three large chromosomal segments not found in the S288C reference genome, called regions A, B and C, on chromosomes VI, XIV and XV, respectively [12]. We looked for these three regions in the genome sequence of 59A, a haploid derivative of EC1118. These three regions are present in the 59A strain, but the 17 kb region B, donated by *Z. bailii*, was found on chromosome X, conflicting with its location on chromosome XIV in the EC1118 diploid genome assembly. An analysis of the number of reads obtained during sequencing of the EC1118 genome was consistent with the presence of one copy of region A and C but suggested that there were three copies of region B (Figure 1). Southern blot hybridization on the chromosomes of EC1118 confirmed that three copies of this region were present in EC1118: the copy initially described on chromosome XIV and two additional copies found in chromosomal bands attributed to chromosomes X and XII (Figure 2). This analysis also confirmed the presence of this region in *Z. bailii* and showed that the 59A strain inherited the copy located on chromosome X.

**Figure 3:**
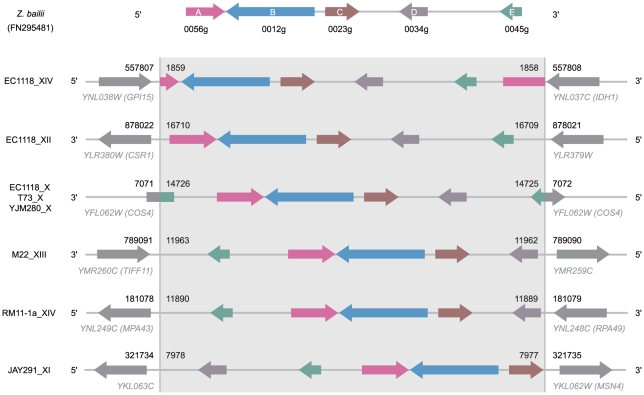
Localization and organization of region B in various strains. Colored arrows represent syntenic ORFs, numbered according to Novo *et al*. [12]. The points of insertion of region B are numbered according to position in the putative circular form proposed in Figure 4. The chromosomal insertion positions are indicated according to the chromosome sequences of S288C.

By carrying out a BLAST similarity search on the sequencing reads of the EC1118 genome, we identified the insertion point on chromosome XII (EC1118_XII form). Region B is located between the *YLR379W* (726 bp downstream from the stop codon) and *YLR380W* (*CSR1,* 261 bp upstream from the start codon) genes, at position 878,021 on S288C chromosome XII (Figure 3). We also characterized the insertion point on chromosome X (EC1118_X form), by analyzing the 59A genome. As previously described for EC1118, the right arm of chromosome X was greatly rearranged [12] Indeed, a 5 kb region from the left arm of chromosome VI encompassing *YFL058W* (*THI5*) and *YFL062W* (*COS4*) was found inserted into the right telomeric end of chromosome X. This rearrangement may have occurred by homologous recombination between two almost identical (98%) *THI* genes (*YJR156C* and *YFL058W*). We found that region B was integrated into the *COS4* gene. Further confirmation of the breakpoint positions on chromosomes XII and X was obtained by PCR amplification with junction-specific primers for EC1118 and 59A.

### Localization and structural organization of B regions in other *S. cerevisiae* strains

We previously showed that region B is present in many *S. cerevisiae* strains, mostly of wine origin [12]. We carried out BLAST similarity searches of the yeast genome sequences available to date. We found that 24 of the 69 yeast genomes checked contained region B (Table S1). As most of these genomes are available as draft assemblies, we could precisely locate the insertion in only five strains (Figure 3). In the vineyard-derived RM11-1a strain, the region is present on the left arm of chromosome XIV, between the *YNL249C* and *YNL248C* genes. The vineyard isolate M22 and the bioethanol production strain derivative JAY291 carry a copy on the right arm of chromosome XIII and on the left arm of chromosome XI, respectively. The T73 strain, a haploid derivative of a commercial wine yeast strain, and YJM280, a clinical isolate, had insertions points similar to those of EC1118_X. We were also able to determine one of the two junctions for four other strains. In the WE372 and CLIB382 strains, the proximal junction is the same as that of M22 on chromosome XIII whereas, for CBS7960 and CLIB324, the proximal junction is identical to that of the EC1118_X form. All forms except EC1118_X, which was found in subtelomeric position, were inserted at internal positions on the chromosome.

Unexpectedly, we also observed variations in gene organization for all these regions. (Figure 3). All five genes were consistently present, but their relative positions varied. The EC1118_XII form exhibits conserved synteny to the *Z. bailii* sequence. For the other forms, the B, C and D genes were syntenic, but the A and E genes varied in position, being located either upstream or downstream from B, C, D group. Genes A and E were sometimes found to have been broken in two, as observed for gene A in the EC1118_XIV form and gene E in the EC1118_X form (Figure 3).

**Figure 4:**
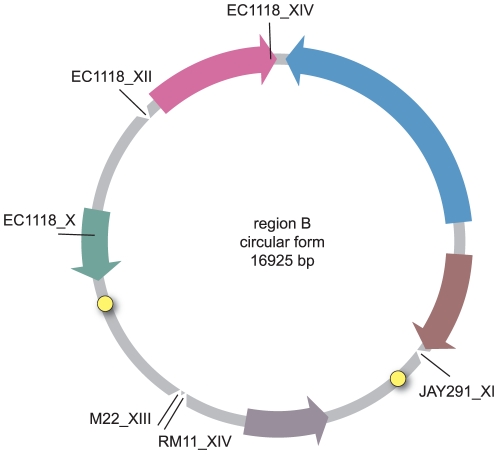
Putative circular form of region B. The insertion points found in various *S. cerevisiae* genomes are indicated by a tick. The two ARS consensus sequence (ACS) are represented by yellow circles.

All these data, including the structural organization of the B regions in the different strains, suggested that that these regions were integrated into the various genomes as closed circular molecules (Figure 4). Furthermore, two copies of an 11 bp ARS consensus sequence (ACS, 5′-WTTTAYRTTTW-3′) were identified in the sequence of region B, at position 8524 to 8535, referring to the circular form, (ACS1, ATTTATATTTT) and 13763 to 13774 (ACS2, TTTTATATTTT). ACS sequences have been identified as essential domains of the ARS element of *S. cerevisiae* [14], [15]. These findings suggest that region B may replicate autonomously in *S*. *cerevisiae.* To address this possibility, we inserted the region encompassing ACS2 in a YIp integrative vector that was then used to transform yeast cells. Transformants were obtained with an efficiency similar to that obtained with a replicative vector, providing evidence that this ARS element is functional in *S. cerevisiae*.

### Analysis of breakpoint sequences

**Figure 5:**
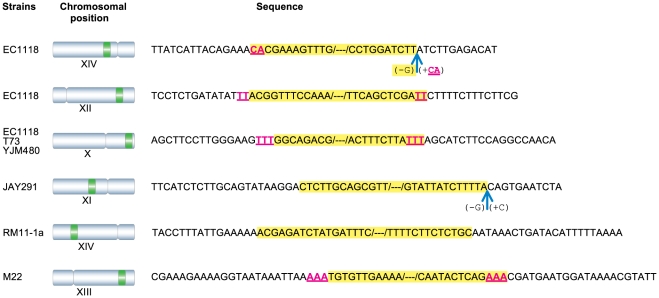
Analysis of the insertion breakpoints in different genomes. The left panel shows the location of region B on the various chromosomes (green boxes). The centromere is represented by a constriction in chromosomes. The right panel shows the chromosomal sequences at the insertion points with the highlighted inserted regions. Deleted and duplicated nucleotides are shown in parentheses, with a minus or plus sign, respectively. Underlined nucleotides indicate sequence identity between the insertion and the chromosomal moieties. All sequences are shown oriented as in the Watson strand of chromosome.

We investigated the mechanisms by which region B had integrated into the genome, by analyzing the nucleotide sequences surrounding the insertion breakpoints in six different chromosomes (Figure 5). The breakpoints and their environment were unique for each insertion. No repetitive elements were found around the breakpoints. At four junctions, a two- to three-nucleotide sequence was found to be common to the sequences of the inserted region and the chromosome (Figure 5). This limited sequence identity suggests the involvement of nonhomologous end joining (NHEJ), a pathway responsible for repairing double-strand breaks in DNA [16]. An analysis of the chromosomal sequences at the breakpoints revealed that the integration of region B was generally not accompanied by changes at the junction site, although a loss or gain of 1 or 2 nucleotides was observed in two cases (Figure 5). We cannot rule out the possibility that these nucleotides are point mutations present on the original chromosome, but they may originate from an addition or deletion event occurring during the repair process, providing further evidence for the role of NHEJ in these insertions.

### Sequence divergence between B regions

**Figure 6:**
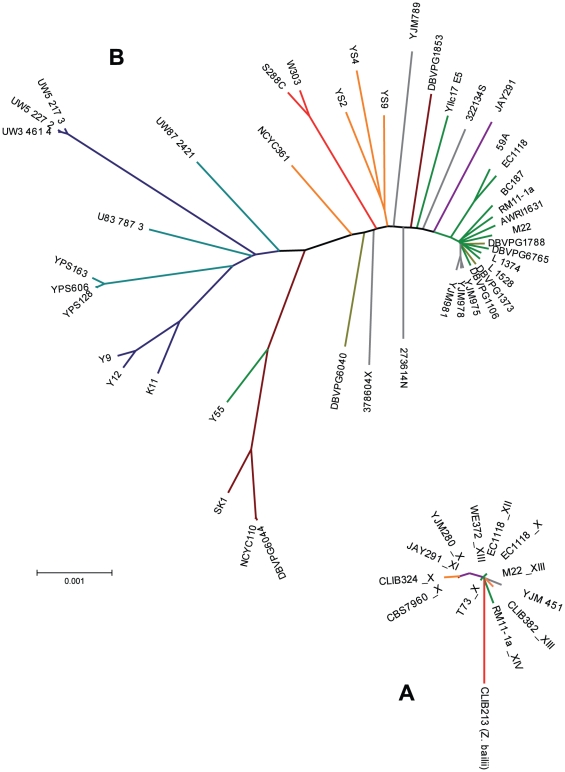
Evolutionary relationships of region B in *Saccharomyces cerevisiae*. A. Neighbor-joining tree based on single nucleotide polymorphism of the region B sequences obtained from *S. cerevisiae* and *Z. bailii*. The labels refer to *S. cerevisiae* strains, followed by the chromosome on which region B was found, if available. B. Neighbor-joining tree based on single nucleotide polymorphism of the genome sequences of 44 *S. cerevisiae* strains. Strains are shown in color according to their technological or geographical origin: clinical isolates in gray, European wine isolates in green, bread isolates in orange, American bioethanol production isolates in purple, European soil isolates in khaki, American isolates in blue-green, Asian isolates in dark blue and African isolates in brown.

We examined the 24 available *S. cerevisiae* genome sequences found to contain region B in more detail (Table S1). Most of these strains belonged to the wine or European group or had been characterized as strains with mosaic genomes [17], [18]. From these genomes, we obtained 10 sequences with sufficient coverage to infer the phylogeny of region B (Figure S1). The resulting dendrogram (Figure 6A) draws a clear picture of the evolution of this region: the *Z. bailii* sequence is in a basal position and presents the longest branch (mean nucleotide divergence of 1.54 substitutions per kb from the group of *S. cerevisiae* strains). By contrast, the *S. cerevisiae* sequences display a low level of diversity, with a mean estimated nucleotide divergence of 0.3 substitutions per kb (Figure 6A), much lower than the diversity between *S. cerevisiae* strains, previously estimated to 1.0–1.4 substitution per kb for wine yeasts and up to 7.3 substitutions per kb for the most distantly related *S. cerevisiae* strains ([17], [19] and Figure 6B). This estimate suggests that the divergence of the region B is at least three time more recent that wine yeast expansion, which was shown to start very likely during or after the Neolithic era [20].

The bioethanol and brewery strains (CLIB324, CBS7960, YJM280 and JAY291) share specific mutations in this region (Figure S1), and have region B inserted at the same chromosomal site, with the exception of JAY291 (Figure 3). Similarly, the B region of strain M22 has a nucleotide sequence identical to that inserted into chromosome XII of EC1118 (EC1118_XII), but the two forms differ in terms of their insertion site. These data suggest that this region can actually move from one locus to another one within the genome.

### Distribution of B regions in *S. cerevisiae* strains of different origins

**Figure 7:**
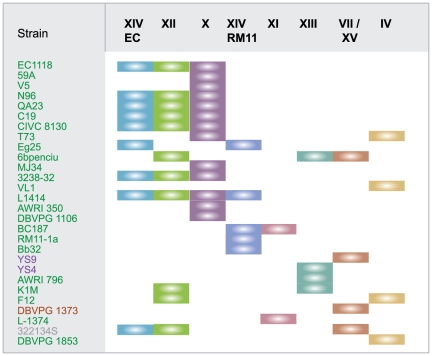
Distribution and localization of region B among yeast strains. PCR and southern blot hybridization were performed on genomic DNA from 28 *S. cerevisiae* strains of different origins, with probes designed to bind to region B, as described in Materials and Methods. The presence of the first five regions was determined by PCR amplification, with primers specifically designed to discriminate between the different forms, and by Southern blot hybridization. The presence of the three last forms was determined by Southern blot hybridization. The origin of the strains is indicated by the color of the name: green for wine, purple for bread, brown for soil and gray for clinical. The distribution of the various forms of region B is represented by colored rectangles: blue for EC1118_XIV, green for EC1118_XII, purple for EC1118_X, dark blue for RM11_XIV, pink for JAY291_XI, blue-green for XIII, brown for VII/XV and gold for IV.

In our previous study [12], we carried out PCR analysis to determine the distribution of region B among 53 *S. cerevisiae* strains of different origins. Region B was found in 25 strains, 20 of which were isolated from the wine environment. Here, we studied the copy number and location of region B in these 25 strains, together with three additional wine yeast strains or derivatives (59A, V5 and N96), through a combination of PFGE, Southern blotting and PCR amplification (Table S1). We obtained evidence for the presence of eight different B regions in these 28 *S. cerevisiae* strains, with up to four different copies present in a single strain, L1414 (Figure 7). All strains closely related to EC1118 (line 59A to 3238-32), with the exception of 6bpenciu, Eg25 and T73, had insertion sites similar to those of EC1118. For two strains (L-1374 and AWRI796) we detected PCR amplifications consistent with the insertions characterized for chromosomes XI and XIII respectively (Figure 3 and Figure 7). However, the successful PCR amplification for the complete set of inter- and intragenic fragments was also consistent with an additional region B. As PFGE and Southern blotting analysis revealed only one chromosomal band, these findings strongly suggest than two copies are present on the same chromosome, separated or in tandem.

## Discussion

We recently described the striking presence of large introgressions from distantly related yeasts in the genome of wine yeasts [12]. We report here that one of these introgressions, the *Z. bailii*–derived 17 kb chromosomal segment, is present in multiple copies in the genome of wine yeasts, mostly at internal positions on various chromosomes (Figure 5). We propose that the amplification and expansion of this fragment in wine yeast has involved the formation of a circle molecule subsequently integrated into the *S. cerevisiae* genome through nonhomologous recombination.

The commercial wine strain EC1118 carries three copies of this region, on three different chromosomes. Similarly, most strains isolated from vineyards or commercial wine yeast strains containing this region carried several copies of it, with up to four copies detected in a given strain. It has been shown that yeast genes transferred from bacteria tend to undergo segmental duplication in their new host [7]. Similarly, the 14-gene chromosomal fragment acquired by intervarietal transfer in the genome of *C. neoformans* is duplicated [10]. The duplication of large DNA segments has occurred repeatedly throughout evolution (see [21]). Intra- and interchromosomal duplications are often mediated by Ty elements, but also occur in the absence of repeated elements, as a result of microhomology/microsatellite-induced replication (MMiR).

Other mechanisms involving extrachromosomal amplification [21], [22], [23], [24], [25], [26] have been reported in natural or experimental yeast populations. For some known cases of extrachromosomal *S. cerevisiae* DNA amplification [23], [25], the presence of a centromere and origins of replication has been reported in amplified fragments. As no genome sequence for *Z. bailii* is currently available, we were unable to determine directly whether replication elements were present. However, within region B, we identified two sequences corresponding to the *S. cerevisiae* ARS consensus sequences (ACS). *S. cerevisiae* ARS elements consist of two essential functional domains: domain A, containing an 11 bp conserved sequence (ACS), and a broad A+T-rich domain B, which flanks domain A but displays no sequence similarity [27]. Although replication origins are not well conserved among eukaryotes, ARS found on plasmids from *Z. rouxii*, *Z. bisporus* and *Z. bailii* were shown to be effective for autonomous replication in *S. cerevisiae* [28], [29], [30], [31]. Therefore, both the structural organization of the integrated regions with a circular permutation of the genes and the presence of two ACS in region B suggest that the circular molecule exists and replicates autonomously in *S. cerevisiae*. This hypothesis was supported by a direct experimental evidence that at least one ARS element is functional in *S. cerevisiae*.

**Figure 8:**
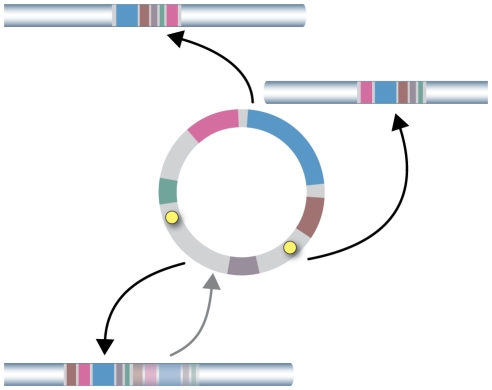
Model of multicopy integration of region B. We propose that the circular form, which can replicates in *S. cerevisiae*, is able to integrate sequentially at different chromosomal locations by non-homologous recombination. In some instance, integration may also occur in tandem and afterwards regenerate an eccDNA molecule by homologous recombination, allowing further integrations (Figure 8).

The mechanism leading to multicopy integration of region B is intriguing. Multiple integrations may have required maintenance of the eccDNA molecule through each sequential integration, before being lost. The finding that an ARS in region B supports autonomous replication in *S. cerevisiae* suggests that the circular form may have been stably maintained for quite a while, resulting in integrations at different locations in the various strains. In addition, the eccDNA molecule may have in some instances integrated in tandem array, as suggested by the detection of two integrations in the same chromosome in two strains. This duplicated molecule might then regenerate an eccDNA molecule by homologous recombination, allowing further integrations (Figure 8).

Most eukaryotic cells have two DNA double-strand break (DSB) repair pathways: homologous recombination (HR) and nonhomologous end joining (NHEJ). The second of these pathways is rare in *S. cerevisiae*, involves the direct rejoining of two DNA molecules and is closely associated with illegitimate recombination and chromosomal rearrangement [16], [32]. The extrachromosomal amplifications described to date in *S. cerevisiae* involve repetitive DNA, as shown for Ty or LTR [23], [25], rDNA [33] and telomeric loci [34]. An analysis of the sequence at breakpoint junctions identified no repeated elements in the immediate vicinity of the insertion points, but showed that integration might have involved microhomology (2 to 3 bases) for four of the six insertions, possibly in association with base mutations in some cases. In NHEJ, the ends of the DNA are joined with little or no base pairing at the junction, and the end-joining product may include small insertions or deletions [35], [36]. Both these features are consistent with our analysis of the sequences at insertion junctions. An alternative mechanism to NHEJ is microhomology-mediated end joining (MMEJ) [37]. However, since the MMEJ mechanism was found to require at least five homologous nucleotides and always leads to deletion or insertions of intervening sequence between the microhomologies [32], [37], [38], the integration of region B by this mechanism seems unlikely.

Random fragments of mitochondrial DNA (NUMTs) can be captured by the nuclear DNA to repair DSB in yeasts [39], [40], [41]. The integration of short fragments of plasmids known as NUPAV has also recently been observed in hemiascomycete yeasts harboring plasmids [42]. It has been suggested that NUMT and NUPAV are formed by occasional aberrant DSB repair events in yeast nuclear DNA [39], [40], [42]. The integration of region B displays certain similarities with that of NUMT and NUPAV: (i) seven of the eight insertion sites observed for region B are intergenic, just as mitochondrial sequences are more frequent in noncoding areas than in coding regions [39]; (ii) no repeated elements were found in the immediate vicinity of B regions, as for 56% of NUMTs [39]. However, one major difference is that no integration of complete molecules has been observed for mitochondrial DNA or for various plasmids. The mechanisms of NUMT formation are unknown, but they result in the presence of random, short (tens to hundreds of nt) mtDNA insertions in the yeast chromosome. By contrast, region B is 17 kb long and is always found intact.

Using a combination of phylogeny and syntheny analyses, we previously showed that region B was acquired by HGT from *Z. bailii*, a wine contaminant, to *S. cerevisiae*, this process probably being facilitated by the proximity of these species in the same ecological niche [12]. This study confirms the transfer from *Z. bailii* and shows that this event occurred after wine strains had begun to diverge, also accounting for this region not being present in all wine strains today. In addition, our data demonstrate, for the first time, the spontaneous amplification of region B in natural wine yeast populations, potentially accounting for its diffusion in wine yeasts and related *S. cerevisiae*. EccDNA have been described in most eukaryotes [43], reflecting the plasticity of the genome. High levels of eccDNA molecules are associated with cell stress or aging, and their formation mostly involves repeated elements, although nonhomologous recombination was reported [43], [44], [45]. It is tempting to speculate that amplification of genes resulting from HGT has helped fermentative *S. cerevisiae* strains to adapt to a new evolutionary niche by providing new or evolved metabolic functions, although the role of the genes carried by region B, encoding a putative oxoprolinase, nicotinamide transporter, Flo11p and transcription factors [12], remains to be determined. We recently identified the function of foreign genes acquired by gene transfer in the genome of EC1118 and of other wine yeast strains [12]: one encodes a high-affinity fructose symporter, providing a new function in *S. cerevisiae* that might confer an adaptive advantage during the fermentation of grape must [13] and two other ones encode oligopeptide transporters [46], which may help yeast cells to assimilate nitrogen at the end of fermentation or after the main fermentation process has been completed. Similarly, as the foreign genes carried on region B were taken up, maintained and expanded in the genome of wine yeast strains, we can infer that they must contribute in some way to increasing the evolutionary fitness of wine yeast.

## Materials and Methods

### Strains

EC1118 (Lalvin EC1118) is a diploid heterozygous commercial wine yeast strain isolated in Champagne (France) and produced and sold commercially by Lallemand Inc. (Canada). Strain 59A was generated from a meiotic haploid spore isolated from EC1118 and selected on the basis of its similar fermentation performance and metabolite production. References for the other yeast isolates are detailed in Table SI. Cells were grown in YPD medium (1% yeast extract, 1% peptone, 1% glucose) at 28°C, with shaking.

### Estimation of the copy number of region B in the EC1118 genome

We used EC1118 sequencing reads to identify changes in copy number along a linear genomic coordinate axis. BLAST [47] was used to align the read sequences to the scaffold EC1118_1N26 (accession no. FN393084). For the sake of clarity, the sequence of the delta Ty2 LTR between positions 21,581 to 21,914 was masked. Each nucleotide position was covered by a mean of 18 reads in the diploid chromosomal region (two copies). Region B was found to be present in three copies, with a mean of 27 reads per nucleotide position.

### Genome sequencing and data analysis

The genome sequence of the wine yeast EC1118 is a “pseudohaploid” assembly of 31 supercontigs [12]. The genome sequence of strain 59A was determined with Illumina Genome Analyzer II technology with 36 bp paired reads (44 X sequencing depth). Velvet software [48] version 0.6.05 was used for *de novo* assembly. The best assembly (*i.e* minimum number of contigs with maximum contig size) was obtained with a hash value of 23, resulting in 2,885 contigs with an N50 size of 11,807 bp. BLAST similarity searches were used to identify contig sequences covering region B of EC1118. Region B was found to encompass two contigs separated by a small gap of 35 bp. This gap was filled by the corresponding sequence from EC1118. The nucleotide sequence of region B from strain 59A was deposited in EMBL-GenBank under accession number HQ615872.

### Southern blot analysis

Southern blot hybridization was performed on yeast chromosomes separated by pulsed-field gel electrophoresis (PFGE), as previously described [49]. Probes were obtained by PCR amplification from EC1118 genomic DNA, using specific primers corresponding to a DNA fragment (gene 0023g) from region B (available upon request). Probes were labeled with the PCR DIG labeling system (Roche Diagnostics), according to the manufacturer's instructions. Chemiluminescence was detected with the CSPD alkaline phosphatase substrate and the DIG Luminescent Detection Kit (Roche Diagnostics).

### Search for region B in *S. cerevisiae* genomes and evolutionary relationships of region B in *Saccharomyces cerevisiae*

We searched for similarity to region B in other *S. cerevisiae* genomes with blastn (no filter). Genome sequences were obtained for 35 strains of the SGRP project of the Sanger Institute [17], 26 strains of the sequencing project at Washington University at St Louis (Justin Fay, http://www.genetics.wustl.edu/jflab/data.html), a bioethanol production yeast derivative JAY291 [50] and a vineyard isolate derivative RM11-1a (*S. cerevisiae* RM11-1a sequencing project, Broad Institute of Harvard and MIT http://www.broad.mit.edu/). We also included in our analysis the sequence of region B from *Z. bailii* [12]. When a significant hit was obtained (expected value <10^−10^, minimum identity 97%), the corresponding sequence was retrieved from contig and low quality regions were clipped if necessary.

All sequences were aligned, with Genious software ver. 4.8.4 (Biomatters Ltd, New Zealand) and MUSCLE software ver. 3.8.31 [51]. Evolutionary history was inferred by the neighbor-joining method [52]. Evolutionary distances were calculated by the Tajima-Nei method [53] and are expressed in the units of the number of base substitutions per site. All positions containing alignment gaps and missing data were eliminated only in pairwise sequence comparisons (pairwise deletion option). Dendrograms were generated and phylogenetic analyses were conducted with MEGA4.1 software [54].

### Genome sequence alignments

Forty four yeast genomes were aligned with MUMmer 3.0 [55], including 35 yeast genomes of the SGRP project [17], JAY291 [50], M22 and YPS163 [56], RM11-1a, YJM789 [57], S288C, AWRI1631 [58], EC1118 [12] and 59A. Repetitive and low-complexity regions that could not be aligned unambiguously were first screened and masked with RepeatMasker (Smit *et al.,* http://www.repeatmasker.org). Polymorphic positions were then extracted, using dedicated Perl scripts to parse the MUMmer output files, with counting of the number of SNPs and indels between the aligned genomes.

### Experimental validation of the junctions of different B regions

Direct experimental support was provided by PCR amplification, with EC1118 or 59A DNA as the template. DNA was isolated as described by Hoffman *et al.* [59]. PCR primers (available upon request) were designed for the specific amplification of region B insertion junctions and conventional chromosomes, similar to those of S288C. The conventional forms were amplified with primers complementary to chromosomal sequences adjacent to the integration site. The region B insertion junctions were amplified with forward and reverse primers complementary to chromosomal sequences adjacent to the integration site and the region B sequence, respectively.

### Test of the autonomous replication function of an ARS element found in region B

The ARS consensus sequence at position 13763 to 13774 (ACS2) was inserted in YIp352 [60], an integrative vector which contains the *S. cerevisiae URA3* gene for selection, at the *Bam*HI site. This ARS amplified fragment was obtained from DNA of 59A strain using the primer pairs: GGATCC ACAGGTTCGAGTAGTTGAT and GGATCC TAGTTCAAGAGGACATGA, corresponding to positions 13596 to 13991 of region B. The *Bam*HI sites were underlined.

The yeast strain CEN.PK2-1C (*MATa*; *ura3-52*; *trp1-289*; *leu2-3,112*; *his3Δ 1*; *MAL2-8^C^*; *SUC2*) (EUROSCARF) was transformed by the LiAC procedure [61]with YIp352, YIp352-ARS and YEp352 [60] as control. Ura^+^ transformants were obtained at a frequency of 4.10^4^ and 1.10^4^ transformants/µg for YIp352-ARS and YEp352 respectively. Extrachromosomal plasmids were recovered from Ura^+^ transformants by transforming *E. coli* to ampicillin resistance. Plasmids were prepared and the ARS region was subsequently sequenced.

### Distribution of B regions in the *S. cerevisiae* population

We tested for the presence of region B variants in various *S. cerevisiae* strains (for a complete list of the strains used, see Table S1), by PCR amplification with primers specifically designed to discriminate between the different forms or by Southern blot hybridization (see above).

## Supporting Information

Figure S1 **Polymorphisms found in region B of** ***Saccharomyces cerevisiae***. The sequence of region B from strain 59A was used to query the available yeast genome sequences. For each strain, the matching sequences (black segments) — often found on different contigs — were used to identify SNP positions (red dots) and indels (green dots). In most cases, the point of integration of the region into the yeast genome was determined (blue bar).
(EPS)

Table S1 **Strains and genomes sequences used in the study.**
(PDF)
