# Supplementary material for: Amplification of a Zygosaccharomyces bailii DNA Segment in Wine Yeast Genomes by Extrachromosomal Circular DNA Formation
Source: PLoS One. 2011 Mar 10;6(3):e17872. doi: 10.1371/journal.pone.0017872 (PMC3053389; doi:10.1371/journal.pone.0017872)
Supplement: Table S1 — Strains and genomes sequences used in the study. (PDF) [file pone.0017872.s002.pdf]

Table S1: Strains and genomes sequences used in the study

Strain name		Gene detection [1]	Chr. Insertion [2]	PFGE detection [3]	BLAST [4]	Origin [5]	Isolation [6]	Provided by	Genome source [7]
Saccharomyces cerevisiae	JAY291				11	Brazil	Haploid derivative from bioethanol production strain PE-2	Sequence provided by GenBank	GenBank:ACFL00000000
Saccharomyces cerevisiae	NT50	-				South Africa	Wine	Anchor Yeast Ltd, South Africa	
Saccharomyces cerevisiae	N96	+	10;12;14a	10;12;14		South Africa	Wine	Anchor Yeast Ltd, South Africa	
Saccharomyces cerevisiae	AWRI1631				+	Australia	Derivative from a meiotic spore of N96	GenBank	GenBank:ABSV00000000
Saccharomyces cerevisiae	AWRI350	+	10	10		Australia	Wine	Australian Wine Research Institute (AWRI), Glen Osmond, Australia	
Saccharomyces cerevisiae	AWRI796	+	-	13 or 16		South Africa	Wine	Australian Wine Research Institute (AWRI), Glen Osmond, Australia	
Saccharomyces cerevisiae	CBS400	-				Ivory Coast	Palm wine	Centraalbureau voor Schimmelcultures (CBS), Utrecht, The Netherlands	
Saccharomyces cerevisiae	CECT1882	-				Spain	Veil from wine	Colección Española de Cultivos Tipo (CECT), Universidad de Valencia, Spain	
Saccharomyces cerevisiae	T73	+	-	4;10		Valencia, Spain	Wine	Departamento de Biotecnología de Alimentos. Instituto de Agroquímica y Tecnología de los Alimentos, Valencia, Spain	
Saccharomyces cerevisiae	MJ104	-				Lebanon	Wine	Centre International de Ressources Microbiennes (CIRM-Levures), Thiverval-Grignon, France	
Saccharomyces cerevisiae	MJ34	+	10	10		Lebanon	Wine	Centre International de Ressources Microbiennes (CIRM-Levures), Thiverval-Grignon, France	
Saccharomyces cerevisiae	MJ73	-				Lebanon	Wine	Centre International de Ressources Microbiennes (CIRM-Levures), Thiverval-Grignon, France	
Saccharomyces cerevisiae	NPA31	-				Nigeria	Palm wine	Dept. Biol. Sciences, Michael Okpara University of Agriculture, Umudike, Abia State, Nigeria	
Saccharomyces cerevisiae	3238-32	+	10;12;14a	10;12;14		Sardinia, Italy	Derived from the Sardinian flor strain A9	Dipartimento di Scienze Ambientali Agrarie e Biotecnologie Agro-alimentari (DISAABA), University of Sassari, Sassari, Italy	
Saccharomyces cerevisiae	A9	-				Sardinia, Italy	Veil from Sardinian Arvisionadu wine	Dipartimento di Scienze Ambientali Agrarie e Biotecnologie Agro-alimentari (DISAABA), University of Sassari, Sassari, Italy	
Saccharomyces cerevisiae	M25	-				Sardinia, Italy	Veil from Sardinian Malvasia di Bosa wine	Dipartimento di Scienze Ambientali Agrarie e Biotecnologie Agro-alimentari (DISAABA), University of Sassari, Sassari, Italy	
Saccharomyces cerevisiae	Fermichamp	-				Alsace, France	Wine	DSM Food Specialties B.V., Delft, The Netherlands	
Saccharomyces cerevisiae	YJM789	-			-	Kansas City, Kansas, USA	Clinical isolate derivative	Duke University Medical Center, Durham, USA	GenBank:AAFW00000000
Saccharomyces cerevisiae	Bb32	+	14b	14		California, USA	Wine	Fred Hutchinson Cancer Research Center, Seattle, Washington, USA	
Saccharomyces cerevisiae	RM11-1a	+	14b	14	14	California, USA	Haploid derivative of Bb32(3)	Fred Hutchinson Cancer Research Center, Seattle, Washington, USA	GenBank:AAEG00000000
Saccharomyces cerevisiae	L2226	-				Rhône valley, France	Wine	Inter Rhône, Avignon, France	
Saccharomyces cerevisiae	Zymaflore VL1	+	-	4		Bordeaux, France	Wine	Laffort, Bordeaux, France	
Saccharomyces cerevisiae	EC1118	+	10;12;14a	10;12;14	10;12;14	Champagne, France	Wine	Lallemand Inc, Montreal, Canada	GenBank:FN393058–FN393060,FN393062–FN393087,FN394216,FN394217
Saccharomyces cerevisiae	QA23	+	10;12;14a	10;12;14		Portugal	Wine	Lallemand Inc, Montreal, Canada	
Saccharomyces cerevisiae	Côte de Blancs	-				France	Wine	Lesaffre , Marcq-en-Baroeul, France	
Saccharomyces cerevisiae	Levuline BRG	-				Burgundy, France	Wine	Oenofrance, Bordeaux, France	
Saccharomyces cerevisiae	C19	+	10;12;14a	10;12;14		Alsace, France	Wine	Oenofrance, Bordeaux, France	
Saccharomyces cerevisiae	CIVC8130	+	10;12;14a	10;12;14		Champagne, France	Wine	Oenofrance, Bordeaux, France	
Saccharomyces cerevisiae	YS2				+	Australia	Baker strain	*Saccharomyces* Genome Resequencing Project (SGRP), University of Nottingham, England	SGRP data download
Saccharomyces cerevisiae	YS4	+	-	13 or 16	+	Netherlands	Baker strain	*Saccharomyces* Genome Resequencing Project (SGRP), University of Nottingham, England	SGRP data download
Saccharomyces cerevisiae	YS9	+	-	7 or 15	+	Singapore	Baker strain	*Saccharomyces* Genome Resequencing Project (SGRP), University of Nottingham, England	SGRP data download
Saccharomyces cerevisiae	DBVPG6044	-			-	West Africa	Bili wine	*Saccharomyces* Genome Resequencing Project (SGRP), University of Nottingham, England	SGRP data download
Saccharomyces cerevisiae	UWOPS87-2421	-			-	Hawaii	Cladode, *Opuntia megacantha*	*Saccharomyces* Genome Resequencing Project (SGRP), University of Nottingham, England	SGRP data download
Saccharomyces cerevisiae	322134S	+	12;14	7 or 15	+	RVI, Newcaslte UK	Clinical isolate	*Saccharomyces* Genome Resequencing Project (SGRP), University of Nottingham, England	SGRP data download
Saccharomyces cerevisiae	378604X				-	RVI, Newcaslte UK	Clinical isolate	*Saccharomyces* Genome Resequencing Project (SGRP), University of Nottingham, England	SGRP data download
Saccharomyces cerevisiae	273614N	-			-	RVI, Newcaslte UK	Clinical isolate	*Saccharomyces* Genome Resequencing Project (SGRP), University of Nottingham, England	SGRP data download
Saccharomyces cerevisiae	YJM978				-	Bergamo, Italy	Clinical isolate	*Saccharomyces* Genome Resequencing Project (SGRP), University of Nottingham, England	SGRP data download
Saccharomyces cerevisiae	YJM981				-	Bergamo, Italy	Clinical isolate	*Saccharomyces* Genome Resequencing Project (SGRP), University of Nottingham, England	SGRP data download
Saccharomyces cerevisiae	YJM975				-	Bergamo, Italy	Clinical isolate	*Saccharomyces* Genome Resequencing Project (SGRP), University of Nottingham, England	SGRP data download
Saccharomyces cerevisiae	UWOPS83-787.3	-			-	Bahamas	Fruit, *Opuntia stricta*	*Saccharomyces* Genome Resequencing Project (SGRP), University of Nottingham, England	SGRP data download
Saccharomyces cerevisiae	NCYC361				+	Ireland	Beer spoilage from wort	*Saccharomyces* Genome Resequencing Project (SGRP), University of Nottingham, England	SGRP data download
Saccharomyces cerevisiae	NCYC110				-	West Africa	Ginger beer from *Z. officinale*	*Saccharomyces* Genome Resequencing Project (SGRP), University of Nottingham, England	SGRP data download
Saccharomyces cerevisiae	UWOPS03-461.4				-	Malaysia	Nectar, Bertam palm	*Saccharomyces* Genome Resequencing Project (SGRP), University of Nottingham, England	SGRP data download
Saccharomyces cerevisiae	UWOPS05-217.3				-	Malaysia	Nectar, Bertam palm	*Saccharomyces* Genome Resequencing Project (SGRP), University of Nottingham, England	SGRP data download
Saccharomyces cerevisiae	YPS606				-	Pennsylvania, USA	Oak	*Saccharomyces* Genome Resequencing Project (SGRP), University of Nottingham, England	SGRP data download
Saccharomyces cerevisiae	YPS128				-	Pennsylvania, USA	Soil beneath oak	*Saccharomyces* Genome Resequencing Project (SGRP), University of Nottingham, England	SGRP data download
Saccharomyces cerevisiae	Y9				-	Japan	Ragi (similar to sake wine)	*Saccharomyces* Genome Resequencing Project (SGRP), University of Nottingham, England	SGRP data download
Saccharomyces cerevisiae	DBVPG6040				-	Netherlands	Fermenting fruit juice	*Saccharomyces* Genome Resequencing Project (SGRP), University of Nottingham, England	SGRP data download
Saccharomyces cerevisiae	SK1	-			-	USA	Soil	*Saccharomyces* Genome Resequencing Project (SGRP), University of Nottingham, England	SGRP data download
Saccharomyces cerevisiae	DBVPG1788	-			-	Finland	Soil	*Saccharomyces* Genome Resequencing Project (SGRP), University of Nottingham, England	SGRP data download
Saccharomyces cerevisiae	DBVPG1373	+	-	7 or 15	+	Netherlands	Soil	*Saccharomyces* Genome Resequencing Project (SGRP), University of Nottingham, England	SGRP data download
Saccharomyces cerevisiae	UWOPS05-227.2	-			-	Malaysia	*Trigona spp* (Stingless bee) collected near Bertam palm flower	*Saccharomyces* Genome Resequencing Project (SGRP), University of Nottingham, England	SGRP data download
Saccharomyces cerevisiae	DBVPG6765	-			-	Unknown	Unknown	*Saccharomyces* Genome Resequencing Project (SGRP), University of Nottingham, England	SGRP data download
Saccharomyces cerevisiae	DBVPG1853	+	-	4	+	Ethiopia	White Teff (*Eragrostis tef*)	*Saccharomyces* Genome Resequencing Project (SGRP), University of Nottingham, England	SGRP data download
Saccharomyces cerevisiae	DBVPG1106	+	10	10	+	Australia	Grapes	*Saccharomyces* Genome Resequencing Project (SGRP), University of Nottingham, England	SGRP data download
Saccharomyces cerevisiae	YIIc17-E5	-			-	Sauternes, France	Wine	*Saccharomyces* Genome Resequencing Project (SGRP), University of Nottingham, England	SGRP data download
Saccharomyces cerevisiae	BC187	+	14b	11;14	+	Napa Valley, USA	Wine	*Saccharomyces* Genome Resequencing Project (SGRP), University of Nottingham, England	SGRP data download
Saccharomyces cerevisiae	L-1374	+	-	11	+	Chile	Wine	*Saccharomyces* Genome Resequencing Project (SGRP), University of Nottingham, England	SGRP data download
Saccharomyces cerevisiae	L-1528				+	Chile	Wine	*Saccharomyces* Genome Resequencing Project (SGRP), University of Nottingham, England	SGRP data download
Saccharomyces cerevisiae	Y55				-	France	Grapes	*Saccharomyces* Genome Resequencing Project (SGRP), University of Nottingham, England	SGRP data download
Saccharomyces cerevisiae	Y12				-	Ivory Coast	Palm wine	*Saccharomyces* Genome Resequencing Project (SGRP), University of Nottingham, England	SGRP data download
Saccharomyces cerevisiae	K11	-			-	Japan	Shochu sake strain	*Saccharomyces* Genome Resequencing Project (SGRP), University of Nottingham, England	SGRP data download
Saccharomyces cerevisiae	W303				-	Unknown	Laboratory	*Saccharomyces* Genome Resequencing Project (SGRP), University of Nottingham, England	SGRP data download
Saccharomyces cerevisiae	L1414	+	10;12;14ab	-		Beaujolais France	Wine	Sicarex Beaujolais, Villefranche sur Saône, France	
Saccharomyces cerevisiae	Vitilevure 58W3	-				Alsace, France	Wine	Sofralab, Epernay, France	
Saccharomyces cerevisiae	K1M	+	12	12;(13 or 16)		Montpellier, France	Wine	UMR1083, Sciences Pour l'Oenologie (SPO), INRA, Montpellier, France	
Saccharomyces cerevisiae	V5	+	10	10		Montpellier, France	Meiotic spore of strain CIVC8130	UMR1083, Sciences Pour l'Oenologie (SPO), INRA, Montpellier, France	
Saccharomyces cerevisiae	59A	+	10	10	10	Montpellier, France	Meiotic spore of strain EC1118	UMR1083, Sciences Pour l'Oenologie (SPO), INRA, Montpellier, France	
Saccharomyces cerevisiae	S288C	-		-	-	Montpellier, France	Laboratory	UMR1083, Sciences Pour l'Oenologie (SPO), INRA, Montpellier, France	*Saccharomyces* Genome Database (SGD)
Saccharomyces cerevisiae	522Davies	-				Burgundy, France	Wine	UMR1131, Santé de la Vigne et Qualité du Vin (SVQV), INRA, Colmar, France	
Saccharomyces cerevisiae	Eg25	+	14ab	14		Alsace, France	Wine	UMR1131, Santé de la Vigne et Qualité du Vin (SVQV), INRA, Colmar, France	
Saccharomyces cerevisiae	Eg8/136	-				Alsace, France	Wine	UMR1131, Santé de la Vigne et Qualité du Vin (SVQV), INRA, Colmar, France	
Saccharomyces cerevisiae	6bPenciu	+	12	(7 or 15);(13 or 16);12		Romania	Wine	University of Bucharest, Romania	
Saccharomyces cerevisiae	F12	+	12	4;12		Cordoba, Spain	Veil from wine	University of Cordoba, Cordoba, Spain	
Saccharomyces cerevisiae	F25	-				Cordoba, Spain	Veil from wine	University of Cordoba, Cordoba, Spain	
Saccharomyces cerevisiae	M22				13	Italy	Vineyard	Washington University School of Medicine in St Louis, USA	Courtesy Justin Fay, Center for Genome Sciences, St Louis, MO (USA)
Saccharomyces cerevisiae	YPS163				-	Lima, Pennsylvania, USA	Oak tree exudates	Washington University School of Medicine in St Louis, USA	Courtesy Justin Fay, Center for Genome Sciences, St Louis, MO (USA)
Saccharomyces cerevisiae	CBS7960 (=DBVPG7960)				10	Sao Paulo, Brazil	Ethanol production factory	Washington University School of Medicine in St Louis, USA	Courtesy Justin Fay, Center for Genome Sciences, St Louis, MO (USA)
Saccharomyces cerevisiae	CLIB215				-	New Zealand	Baker's yeast	Washington University School of Medicine in St Louis, USA	Courtesy Justin Fay, Center for Genome Sciences, St Louis, MO (USA)

Saccharomyces cerevisiae	CLIB324				10	Saigon, Vietnam	Baker's yeast	Washington University School of Medicine in St Louis, USA	Courtesy Justin Fay, Center for Genome Sciences, St Louis, MO (USA)
Saccharomyces cerevisiae	CLIB382 (=DBVPG6175)				13	Ireland	Super-attenuated beer	Washington University School of Medicine in St Louis, USA	Courtesy Justin Fay, Center for Genome Sciences, St Louis, MO (USA)
Saccharomyces cerevisiae	FL100 (= ATCC28383)				-		Laboratory	Washington University School of Medicine in St Louis, USA	Courtesy Justin Fay, Center for Genome Sciences, St Louis, MO (USA)
Saccharomyces cerevisiae	I14				-	Petrina, Italy	Soil	Washington University School of Medicine in St Louis, USA	Courtesy Justin Fay, Center for Genome Sciences, St Louis, MO (USA)
Saccharomyces cerevisiae	IL-01				-	Cahokia, Illinois	Soil	Washington University School of Medicine in St Louis, USA	Courtesy Justin Fay, Center for Genome Sciences, St Louis, MO (USA)
Saccharomyces cerevisiae	NC-02				-	North Caroline, USA	Oak tree exudates	Washington University School of Medicine in St Louis, USA	Courtesy Justin Fay, Center for Genome Sciences, St Louis, MO (USA)
Saccharomyces cerevisiae	PW5				-	Aba, Abia state, Nigeria	Raphia palm wine	Washington University School of Medicine in St Louis, USA	Courtesy Justin Fay, Center for Genome Sciences, St Louis, MO (USA)
Saccharomyces cerevisiae	T7				-	Babler State Park, Missouri, USA	Oak tree exudates	Washington University School of Medicine in St Louis, USA	Courtesy Justin Fay, Center for Genome Sciences, St Louis, MO (USA)
Saccharomyces cerevisiae	T73				10	Alicante, Spain	Wine	Washington University School of Medicine in St Louis, USA	Courtesy Justin Fay, Center for Genome Sciences, St Louis, MO (USA)
Saccharomyces cerevisiae	UC5				-	Kurashi, Japan	Sene sake	Washington University School of Medicine in St Louis, USA	Courtesy Justin Fay, Center for Genome Sciences, St Louis, MO (USA)
Saccharomyces cerevisiae	WE372				13	Cape Town, South Africa	Wine	Washington University School of Medicine in St Louis, USA	Courtesy Justin Fay, Center for Genome Sciences, St Louis, MO (USA)
Saccharomyces cerevisiae	Y10 (=NRRL y7567)				-	Phillipines	Coconut	Washington University School of Medicine in St Louis, USA	Courtesy Justin Fay, Center for Genome Sciences, St Louis, MO (USA)
Saccharomyces cerevisiae	Y12 (=NRRL y12633)				-	Ivory Coast	Palm wine	Washington University School of Medicine in St Louis, USA	Courtesy Justin Fay, Center for Genome Sciences, St Louis, MO (USA)
Saccharomyces cerevisiae	Y9 (=NRRL y5997)				-	Java, Indonesia	Ragi (African or finger millet)	Washington University School of Medicine in St Louis, USA	Courtesy Justin Fay, Center for Genome Sciences, St Louis, MO (USA)
Saccharomyces cerevisiae	YJM269				-		Grapes	Washington University School of Medicine in St Louis, USA	Courtesy Justin Fay, Center for Genome Sciences, St Louis, MO (USA)
Saccharomyces cerevisiae	YJM280				10	USA	Clinical sample	Washington University School of Medicine in St Louis, USA	Courtesy Justin Fay, Center for Genome Sciences, St Louis, MO (USA)
Saccharomyces cerevisiae	YJM320				-	USA	Clinical sample	Washington University School of Medicine in St Louis, USA	Courtesy Justin Fay, Center for Genome Sciences, St Louis, MO (USA)
Saccharomyces cerevisiae	YJM326				-	USA	Clinical sample	Washington University School of Medicine in St Louis, USA	Courtesy Justin Fay, Center for Genome Sciences, St Louis, MO (USA)
Saccharomyces cerevisiae	YJM421				-	USA	Clinical sample	Washington University School of Medicine in St Louis, USA	Courtesy Justin Fay, Center for Genome Sciences, St Louis, MO (USA)
Saccharomyces cerevisiae	YJM428				-	USA	Clinical sample	Washington University School of Medicine in St Louis, USA	Courtesy Justin Fay, Center for Genome Sciences, St Louis, MO (USA)
Saccharomyces cerevisiae	YJM451				+	Europe	Clinical sample	Washington University School of Medicine in St Louis, USA	Courtesy Justin Fay, Center for Genome Sciences, St Louis, MO (USA)
Saccharomyces cerevisiae	YJM653				-		Clinical sample	Washington University School of Medicine in St Louis, USA	Courtesy Justin Fay, Center for Genome Sciences, St Louis, MO (USA)
Saccharomyces cerevisiae	YPS1009				-	Mettler Woods, New Jersey, USA	Oak tree exudates	Washington University School of Medicine in St Louis, USA	Courtesy Justin Fay, Center for Genome Sciences, St Louis, MO (USA)
Zygosaccharomyces bailii	CLIB213^{T} (=CBS680)	+		+		Japan	Brewery	Centre International de Ressources Microbiennes (CIRM-Levures), Thiverval-Grignon, France	

Legend

Positive detection	+
Negative detection	-
Not tested	

Footnotes

[1] Detection of the genes of region B was performed by specific PCR amplification of coding sequences and intergenic regions.

[2] Position on chromosome was tested by specific PCR amplifications of the 4 major junctions present on chromosome 10, 12, 14a (EC1118 form) and 14b (RM11-1a form)

[3] Chromosome identification was assayed by Pulsed Field Gel Electrophoresis (PFGE) followed by Southern blot hybridization using region B's specific probes.

[4] Similarity search of region B in available *S. cerevisiae* genomes was performed using blastn (no filter, expected value < 10^{-10}, minimum identity 97%).

[5] Geographical location of their isolation.

[6] Material from which the strain was isolated.

[7] Origin of the genome sequences:

EMBL-GenBank

Saccharomyces Genome Database (SGD): http://downloads.yeastgenome.org

SGRP data download: ftp://ftp.sanger.ac.uk/pub/dmc/yeast/latest (2008)

Courtesy Justin Fay, Center for Genome Sciences, St Louis, MO (USA): http://www.genetics.wustl.edu/jflab/data.html (October 2010)
